# Risk factors and survival outcomes in patients with breast cancer and lung metastasis: a population‐based study

**DOI:** 10.1002/cam4.1370

**Published:** 2018-02-23

**Authors:** Weikai Xiao, Shaoquan Zheng, Peng Liu, Yutian Zou, Xinhua Xie, Ping Yu, Hailin Tang, Xiaoming Xie

**Affiliations:** ^1^ Department of Breast Oncology State Key Laboratory of Oncology in South China Collaborative Innovation Center for Cancer Medicine Sun Yat‐sen University Cancer Center 651 East Dongfeng Road Guangzhou 510060 China

**Keywords:** Breast cancer, incidence, lung metastases, prognosis

## Abstract

The risk factors for morbidity and mortality in breast cancer lung metastases (BCLM) patients still remain poorly identified. The aim of this study was to assess the incidence and survival of BCLM and associated risk factors. Patients with BCLM were identified from the Surveillance, Epidemiology, and End Results (SEER) database. Multivariate logistic regression analysis was used to determine the risk factors for BCLM. Predictors of factors associated with death were analyzed in Cox regression and Fine and Gray's test. Of the 11568 patients with stage IV breast cancer, 4213 (36.4%) had BCLM and 1214 (10.5%) had metastases confined to lungs. The median survival time for patients with BCLM was 21 months, and 15.5% of the patients were alive more than 3 years. The tumor subtype distribution was 45.3% HR^−^/HER2^−^, 12.2% HR^+^/HER2^+^, 7.8% HR^−^/HER2^+,^ and 15.0% triple‐negative subtype. Compared with patients without BCLM, those with BCLM were more likely to be aged, female, black, higher tumor grade, HR^−^/HER2^+^, HR^+^/HER2^+,^ and triple‐negative subtypes at diagnosis. Survival analysis showed that the aged, black race, HR^−^/HER2^+^, triple‐negative subtype, higher grade were the independent risk factor for BCLM patients’ survival, while HR^+^/HER2^+^ subtype, insured status, and married status suggested better prognosis. In conclusion, the incidence and prognosis of BCLM varied by tumor subtypes, age, and race. Elderly patients with HER2‐positive or triple‐negative tumors were more likely to have BCLM.

## Introduction

Lung metastasis is one of the most common distant metastases of breast cancer 1, 2. In autopsy studies on metastatic patterns of breast cancer, 57–77% of patients were found to have lung metastases 3. In a retrospective analysis of 1581 patients with metastatic breast cancer, about 23% of patients had lung metastases, 5.6% of patients with metastases confined to lungs 4. It is reported that for breast cancer patients with metastases confined to lungs, the median overall survival (OS) after systemic chemotherapy was 22.5 months 5. Therefore, the development of lung metastasis in breast cancer is associated with poor prognosis. However, it is unclear which factors affect the morbidity and mortality of BCLM. Tumor subtypes are associated with metastatic pattern of metastatic breast cancer. Previous studies have reported that the probability of lung recurrence in patients with early breast cancer with hormone receptor (HR)‐positive human epidermal growth factor receptor 2 (HER2)‐positive and triple‐negative subtypes was higher than HR‐positive HER2‐negative tumors 6, 7, 8, 9, 10, 11. However, the effect of tumor subtypes on BCLM survival is unclear. In addition, the impact of sociodemographic factors and clinical characteristics on the incidence and mortality of BCLM has not been adequately studied.

Better understanding of the incidence and survival of BCLM and related risk factors can help identify patients with high‐risk factors, and reduce the occurrence of BCLM and improve the prognosis by early intervention. The aim of this study was to assess the incidence and survival of BCLM and its associated risk factors.

## Materials and Methods

### Patients

We extracted data from 18 registries released in 2016 (the latest follow‐up information available) within the Surveillance, Epidemiology, and End Results (SEER) database which contains the limited medical information for about 30% of the total American population 12. Using SEER*Stat (version 8.3.4 National Cancer Institute, Bethesda, MD, USA), we attained a cohort of 247,364 patients diagnosed as primary and histologically validated malignant breast cancer and aged 18 years or above at diagnosis from January 1st, 2010 to December 31st, 2013. Those with carcinoma in situ were excluded in this cohort. Furthermore, we generated a final incidence cohort of 240,808 patients with definite lung metastases status at diagnosis (Yes or No). Within the case listing, 4213 patients had lung metastases when first diagnosed as having breast cancer. Subsequently, we excluded patients who were diagnosed by autopsy or death certificate and whose survival record presented with 0 month, leaving a survival cohort of 3772 patients with active follow‐up for survival analysis. Before initiating this study, we signed and submitted a data agreement form to the SEER research team, thus having access to SEER database according to official permission. The study was approved by the Institutional Review Board of Sun Yat‐sen University Cancer Center, Guangzhou, Guangdong Province, People's Republic of China. The review board waived informed consent from patients because of unknown identity.

### Stratification

Incidence proportion was defined as the number of patients with lung metastases divided by the number of patients with breast cancer. We calculated the absolute quantity and incidence proportion of patients with lung metastases confirmed at breast cancer diagnosis among the entire cohort and metastatic diseases subgroup after breast cancer molecular subtype stratification which includes hormone receptor (HR)‐positive human epidermal growth factor receptor 2 (HER2)‐positive, HR^−^/HER2^+^, HR^+^/HER2^−,^ and triple‐negative (HR‐negative HER2‐negative), respectively. Patients were also stratified by age, race, sex, marital status, number of metastatic sites outside of the lungs, pathological grade, etc. Race/ethnicity was comprised of white, black, Asian or Pacific Islander, and American Indian/Alaska Native in accord with the database record.

### Statistical analysis

To assess the correlation between variables and lung metastases status, we used multivariate logistic regression model to calculate the odds ratios (ORs) within the subgroups, adjusted for all variables which may harbor different prognosis. The extent of metastatic diseases was characterized by the presence or absence of brain, liver, and bone metastases available in the SEER database.

The survival was defined as the time from the initial breast cancer diagnosis to death. We used Kaplan–Meier method to compute the survival estimates and generate survival curves within subsets of subtypes and overall cohort. A Cox proportional hazards regression analysis was conducted to assess the association of the same variables described herein with the hazard ratio (HR) of death in patients with lung metastases. Fine and Gray's semiparametric competing risk model was used to exam the subdistribution hazards.

We calculated 95% confidence intervals (95% CI) for all estimates (ORs and HRs) across strata. A *P* value of 0.05 or less was determined as statistically significant. All *P* values were two‐tailed. Statistical analysis was performed using SPSS statistical software (SPSS IBM STATISTICS 21, IBM Corporation, Armonk, USA), apart from the Kaplan–Meier method by SAS 9.4 software (SAS Institute, Cary, NC) and breast cancer‐specific mortality using a Fine and Gray's semiparametric model by cmprsk package of R software (version 3.4.1 R Foundation).

## Results

The absolute quantity and incidence proportion of our cohort according to molecular subtypes appear in Table 1. The incidence proportion of HR^+^/HER2^−^, HR^+^/HER2^+^, HR^−^/HER2^+^, triple‐negative, and unknown subtypes among 240,808 patients diagnosed with malignant breast cancer between 2010 and 2013 was 67.7%, 9.3%, 4.1%, 10.7%, and 8.3%, respectively. Among the 11,568 patients diagnosed with metastatic diseases at distant sites analyzed for incidence, 51.8%, 13.2%, 6.9%, 11.1%, and 17.0% had HR^+^/HER2^−^, HR^+^/HER2^+^, HR^−^/HER2^+^, triple‐negative, and unknown subtypes, respectively. Four thousand two hundred and thirteen lung metastatic patients were identified, accounting for 1.8% and 36.4% of the entire study cohort and subgroup with distant metastases, respectively. Of these, 1214 were patients with metastases confined to lungs (i.e., metastatic disease only in the lungs). The patients with HR^+^/HER2^−^ subtype harbored the highest incidence proportion (3.4% of the entire study population, 42.4% of the metastatic subgroup) and HR^+^/HER2^−^ subtype (1.2% of the entire study population, 31.8% of the metastatic subgroup) the lowest.

**Table 1:** The incidence and median survival of patients with lung metastases from breast cancer stratified by subtypes

Subtype	Patients, No.			Incidence proportion of lung metastasis, %		Median survival of patients with lung metastases (IQR), months
	With breast cancer	With metastatic diseases	With lung metastases	Among entire cohort	Among subgroup with metastatic diseases	
HR^+^/HER2^−^	162,952	5993	1907	1.2	31.8	28.0 (10.0–59.0)
HR^+^/HER2^+^	22,455	1529	512	2.3	33.5	31.0 (15–NR)
HR^−^/HER2^+^	9840	798	330	3.4	41.4	21.0 (7.0–46.0)
Triple‐negative	25,638	1284	633	2.5	49.3	11.0 (4.0–20.0)
Unknown	19,923	1964	831	4.2	42.3	12.0 (3.0–34.0)
All subtypes	240,808	11,568	4213	1.8	36.4	21.0 (7.0–48.0)

HER2, human epidermal growth factor receptor 2; HR, hormone receptor; IQR, interquartile range; NR, not reached. + Denotes positive; − denotes negative.

The multivariable logistic regression results after the stratification of demographic and clinical characteristics are provided in Table 2. Among the metastatic subgroup, age 40–59 years (vs. age 18–39 years; OR, 1.36; 95% CI: 1.13–1.63; *P *= 0.001), age 60–79 years (vs. age 18–39 years; OR, 1.85; 95% CI: 1.54–2.22; *P *< 0.001), age ≥80 years (vs. age 18–39 years; OR, 2.13; 95% CI: 1.73–2.62; *P < *0.001), male (vs. female; OR, 1.85; 95% CI: 1.29–2.65; *P *= 0.001), black race (vs. white race; OR, 1.19; 95% CI, 1.07–1.33; *P *= 0.001), two metastatic sites outside of lungs (vs. 0 or 1 site; OR, 1.30; 95% CI: 1.18–1.44; *P* < 0.001) and three metastatic sites outside of lungs (vs. 0 or 1 site; OR, 3.76; 95% CI: 2.84–4.98; *P* < 0.001), HR^−^/HER2^+^ (vs. HR^+^/HER2^−^; OR, 1.37; 95% CI: 1.17–1.60; *P* < 0.001), triple‐negative subtypes (vs. HR^+^/HER2^−^; OR, 1.82; 95% CI: 1.60–2.07; *P *< 0.001), pathological grade II (vs. grade I; OR, 2.93; 95% CI: 2.50–3.44; *P* < 0.001), III (vs. grade I; OR, 4.77; 95% CI: 4.06–5.62; *P* < 0.001) and IV (vs. grade I; OR, 8.41; 95% CI: 5.92–11.94; *P* < 0.001) were more likely to be diagnosed as lung metastases at initial diagnosis. Interestingly, married (vs. unmarried; OR, 0.85; 95% CI: 0.78–0.92; *P *< 0.001) and insured (vs. uninsured; OR, 0.64; 95% CI: 0.54–0.77; *P *< 0.001) status seemed to be associated with lower odds of lung metastases at diagnosis. The results among the entire study population reflected a similar trend. Significant results appear in Table 2.

**Table 2:** Multivariate logistic regression of lung metastases

Variables	Patients, No		Among entire cohort		Among subset with metastatic diseases	
	Patients (*n* = 240,808)	With lung metastases (*n* = 4213)	OR (95% CI)	*P* value	OR (95% CI)	*P* value
Subtype						
HR^+^/HER2^−^	162,952	1907	1 (Reference)		1 (Reference)	
HR^+^/HER2^+^	22,455	512	1.27 (1.14–1.42)	0.007	1.02 (0.90–1.15)	0.791
HR^−^/HER2^+^	9840	330	1.62 (1.41–1.85)	<0.001	1.37 (1.17–1.60)	<0.001
Triple‐negative	25,638	633	1.42 (1.27–1.57)	<0.001	1.82 (1.60–2.07)	<0.001
Unknown	19,923	831	1.63 (1.48–1.81)	<0.001	1.39 (1.24–1.56)	<0.001
Age at diagnosis, year^a^						
18–39	11,225	187	1 (Reference)		1 (Reference)	
40–59	95,084	1423	1.18 (1.00–1.39)	0.055	1.36 (1.13–1.63)	0.001
60–79	107,713	1950	1.68 (1.42–1.98)	<0.001	1.85 (1.54–2.22)	<0.001
≥80	26,784	653	2.02 (1.69–2.42)	<0.001	2.13 (1.73–2.62)	<0.001
Sex						
Female	238,934	4151	1 (Reference)		1 (Reference)	
Male	1874	62	1.96 (1.48–2.59)	<0.001	1.85 (1.29–2.65)	0.001
Race						
White	191,790	3092	1 (Reference)		1 (Reference)	
Black	26,612	792	1.40 (1.28–1.53)	<0.001	1.19 (1.07–1.33)	0.001
Asian or Pacific Islander	19,479	283	0.98 (0.86–1.12)	0.739	1.31 (1.12–1.54)	0.001
American Indian/Alaska Native	1372	31	1.50 (1.02–2.20)	0.038	1.58 (0.97–2.57)	0.066
Unknown	1555	15	0.53 (0.31–0.91)	0.021	1.09 (0.57–2.10)	0.791
Marital status						
Unmarried	98,180	2361	1 (Reference)			
Married	128,665	1599	0.65 (0.60–0.70)	<0.001	0.85 (0.78–0.92)	<0.001
Unknown	13,963	253	0.77 (0.66–0.89)	<0.001	1.01 (0.85–1.20)	0.945
Insurance status						
Uninsured	4495	258	1 (Reference)		1 (Reference)	
Insured	231,362	3835	0.42 (0.36–0.49)	<0.001	0.64 (0.54–0.77)	<0.001
Unknown	4951	120	0.47 (0.36–0.49)	<0.001	0.83 (0.61–1.12)	0.224
Number of metastatic sites to bone, brain and liver						
0 or 1	237,899	2974	1 (Reference)		1 (Reference)	
2	2106	832	35.03 (31.65–38.77)	<0.001	1.30 (1.18–1.44)	<0.001
All 3	228	148	102.98 (76.90–137.92)	<0.001	3.76 (2.84–4.98)	<0.001
Unknown	575	259	39.40 (32.84–47.27)	<0.001	3.68 (2.96–4.58)	<0.001
Pathological Grade						
1	51,553	177	1 (Reference)		1 (Reference)	
2	98,969	1192	2.93 (2.50–3.44)	<0.001	1.40 (1.16–1.69)	<0.001
3	73,236	1680	4.77 (4.06–5.62)	<0.001	1.86 (1.54–2.24)	<0.001
4	1101	48	8.41 (5.92–11.94)	<0.001	2.55 (1.62–4.01)	<0.001
Unknown	15,949	1116	10.16 (8.57–12.04)	<0.001	1.42 (1.17–1.73)	<0.001

HER2, human epidermal growth factor receptor 2; HR, hormone receptor; OR, odds ratio; 95% CI: 95% confidence interval; + Denotes positive; − denotes negative.

a Unknown age was removed from model owing to nonconvergence.

### Survival

**Figure 1:**
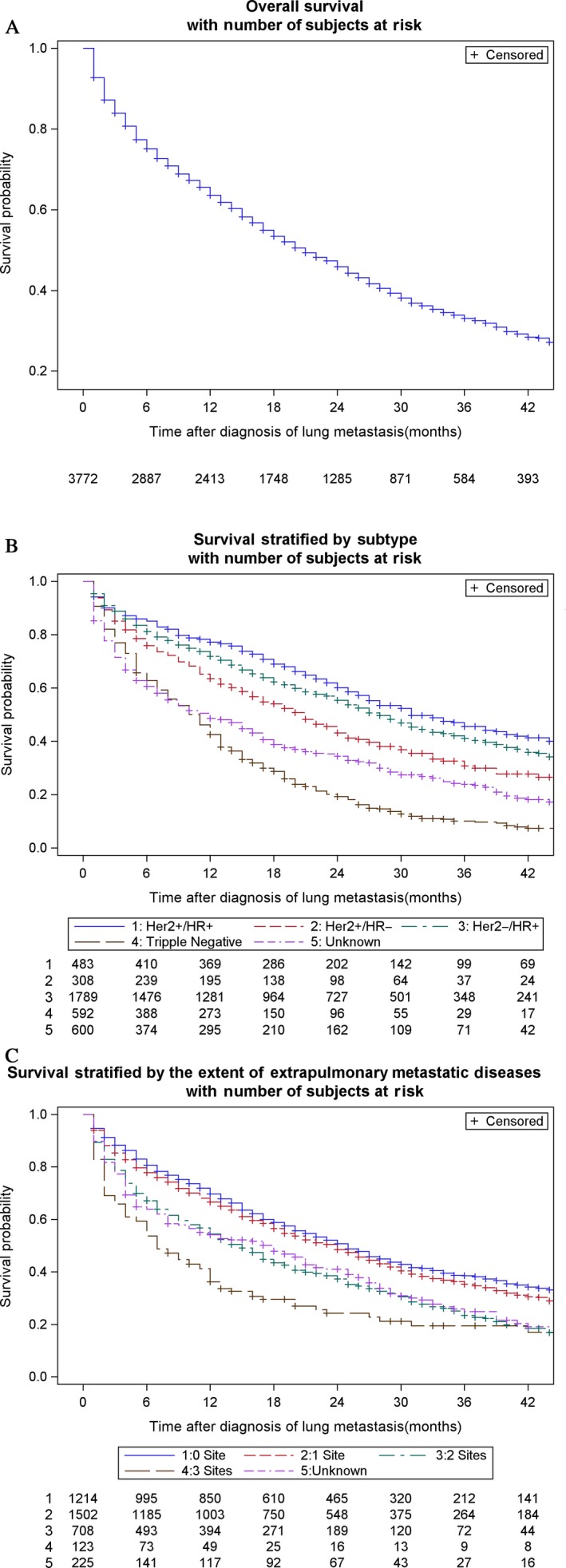
Overall survival and subtype‐stratified survival among patients with lung metastases from breast cancer. The overall survival (A), survival stratified by subtype (B), and survival stratified by the extent of extrapulmonary metastatic disease (C). HER2, human epidermal growth factor receptor 2; HR, hormone receptor. + Denotes positive; − denotes negative.

In the survival cohort of 3772 patients diagnosed as lung metastases, the median survival stratified by subtypes is provided in Table 1. The median survival among the cohort was 21 months, of which the median survival of patients with metastases confined to lungs was 25 months. Patients with HR^+^/HER2^+^ had the longest median survival (31 months) and triple‐negative the shortest (11 months). Figure shows the overall survival (Fig. 1A), survival stratified by subtype (Fig. 1B).

The hazard ratios for all‐cause mortality according to all variables in multivariate Cox regression model appear in Table 3. Age 40–59 years (vs. age 18–39 years; hazard ratio, 1.36; 95% CI: 1.09–1.70; *P *=* *0.006), age 60–79 years (vs. age 18–39 years; hazard ratio, 1.79; 95% CI: 1.44–2.23; *P *< 0.001), and age >80 years (vs. age 18–39 years; hazard ratio, 3.03; 95% CI: 2.39–3.84; *P *< 0.001), black race (vs. white; hazard ratio, 1.24; 95% CI: 1.12–1.38; *P* < 0.001), extrapulmonary metastatic diseases to one site (vs. 0 site; hazard ratio, 1.39; 95% CI: 1.26–1.54; *P* < 0.001), two sites (vs. 0 site; hazard ratio, 2.18; 95% CI: 1.94–2.46; *P* < 0.001), three sites (vs. 0 site; hazard ratio, 3.01; 95% CI: 2.42–3.76; *P* < 0.001), HR^−^/HER2^+^ subtype (vs. HR^+^/HER2^−^ subtype; OR, 1.30; 95% CI: 1.11–1.53; *P* = 0.001), triple‐negative subtype (vs. HR^+^/HER2^−^ subtype; OR, 2.45; 95% CI: 2.18–2.76; *P *< 0.001), pathological grade III (vs. grade I; hazard ratio, 1.73; 95% CI: 1.36–2.21; *P* < 0.001) and IV (vs. grade I; hazard ratio, 2.12; 95% CI: 1.38–3.25; *P* = 0.001) were significantly associated with a increased all‐cause mortality. Married status (vs. unmarried; hazard ratio, 0.79; 95% CI: 0.72–0.86; *P* < 0.001) and HR^+^/HER2^+^ subtype (vs. HR^+^/HER2^−^ subtype; OR, 0.82; 95% CI: 0.70–0.94; *P *= 0.001) were significantly associated with decreased all‐cause mortality. But, insured status was not associated with mortality in this model. Breast cancer‐specific mortality of patients with lung metastases at initial diagnosis also appears in Table 3. Median survival of subtypes after the stratification of the extent of metastatic sites is provided in Table 4. Survival was better among those with less metastatic diseases at distant sites. In general, patients with lung metastases at diagnosis experienced significantly shorter survival than patients presented with no baseline lung involvement (Table 4).

**Table 3:** Multivariate Cox regression of all‐cause mortality and specific breast cancer mortality in patients with lung metastasis

Variables	Patients, No		All‐cause mortality		Breast cancer‐special mortality	
	Patients (*n* = 238,781)	With lung metastases (*n* = 3772)	Hazard ratio (95% CI)	*P* value	Hazard ratio (95% CI)	*P* value
Subtype						
HR^+^/HER2^−^	162,162	1789	1 (Reference)		1 (Reference)	
HR^+^/HER2^+^	22,316	483	0.82 (0.70–0.94)	0.006	0.81 (0.69–0.94)	0.007
HR^−^/HER2^+^	9760	308	1.30 (1.11–1.53)	0.001	1.22 (1.03–1.44)	0.025
Triple‐negative	25,467	592	2.45 (2.18–2.76)	<0.001	2.22 (1.96–2.52)	<0.001
Unknown	19,076	600	1.69 (1.50–1.91)	<0.001	1.98 (1.74–2.25)	<0.001
Age at diagnosis, year^a^						
20–39	11,171	178	1 (Reference)		1 (Reference)	
40–59	94,524	1320	1.36 (1.09–1.70)	0.006	1.39 (1.12–1.72)	0.003
60–79	106,851	1740	1.79 (1.44–2.23)	<0.001	1.67 (1.34–2.07)	<0.001
≥80	26,235	534	3.03 (2.39–3.84)	<0.001	2.39 (1.87–3.04)	<0.001
Sex						
Female	236930	3714	1 (Reference)		1 (Reference)	
Male	1851	58	0.77 (0.53–1.11)	0.165	0.66 (0.41–1.05)	0.079
Race						
White	190,352	2770	1 (Reference)		1 (Reference)	
Black	26,345	711	1.24 (1.12–1.38)	<0.001	1.17 (1.03–1.32)	0.015
Asian or Pacific Islander	19,300	251	0.95 (0.80–1.13)	0.558	0.87 (0.71–1.07)	0.18
American Indian/Alaska Native	1361	28	0.98 (0.62–1.54)	0.93	1.17 (0.73–1.89)	0.51
Unknown	1423	12	0.51 (0.19–1.37)	0.183	0.58 (0.24–1.44)	0.24
Marital status						
Unmarried	97,131	2085	1 (Reference)		1 (Reference)	
Married	128,031	1473	0.79 (0.72–0.86)	<0.001	0.81 (0.73–0.89)	<0.001
Unknown	13,637	214	0.72 (0.59–0.87)	0.001	0.68 (0.55–0.85)	<0.001
Insurance status						
Uninsured	4347	207	1 (Reference)		1 (Reference)	
Insured	229,751	3467	0.85 (0.71–1.02)	0.081	0.91 (0.74–1.11)	0.36
Unknown	4683	98	0.90 (0.65–1.24)	0.504	0.98 (0.67–1.43)	0.90
Number of metastatic sites to bone, brain and liver						
0	229,105	1214	1 (Reference)		1 (Reference)	
1	7104	1502	1.39 (1.26–1.54)	<0.001	1.40 (1.24–1.57)	<0.001
2	1860	708	2.18 (1.94–2.46)	<0.001	2.30 (2.00–2.64)	<0.001
3	193	123	3.01 (2.42–3.76)	<0.001	3.20 (2.44–4.21)	<0.001
Unknown	519	225	1.60 (1.34–1.91)	<0.001	1.65 (1.32–2.06)	<0.001
Pathological Grade						
1	51,324	166	1 (Reference)		1 (Reference)	
2	98,432	1113	1.22 (0.96–1.56)	0.107	0.79 (0.67–0.93)	<0.001
3	73,736	1562	1.73 (1.36–2.21)	<0.001	1.22 (1.02–1.47)	<0.03
4	1083	42	2.12 (1.38–3.25)	0.001	2.38 (2.07–2.73)	<0.001
Unknown	15,206	889	1.56 (1.22–2.00)	<0.001	1.72 (1.48–2.00)	<0.001

HER2, human epidermal growth factor receptor 2; HR, hormone receptor; OR, odds ratio; 95% CI: 95% confidence interval; + Denotes positive; − denotes negative.

a Unknown age was removed from model due to nonconvergence.

**Table 4:** The median survival of patients with breast cancer stratified by sites of metastases

Subtype	Type of metastasis	Survival, median(IQR), months		
		Without lung metastasis	With lung metastasis	Log Rank *P* value
HR^+^/HER2^−^	Bone	36.0 (18.0–NR)	26.0 (9.0–53.0)	<0.001
	Liver	24.0 (9.0–46.0)	17.0 (5.0–41.0)	0.011
	Brain	13.0 (5.0–35.0)	17.0 (4.0–40.0)	0.340
	2 of 3	22.0 (8.0–41.0)	17.0 (6.0–40.0)	0.157
	All 3	10.0 (3.0–22.0)	16.0 (4.0–52.0)	0.081
HR^+^/HER2^+^	Bone	45.0 (21.0–NR)	31.0 (12.0–NR)	<0.001
	Liver	41.0 (17.0–NR)	22.0 (6.0–NR)	<0.001
	Brain	34.0 (12.0–NR)	17.0 (5.0–40.0)	0.024
	2 of 3	34.0 (16.0–NR)	22.0 (6.0–NR)	0.002
	All 3	15.0 (3.0–NR)	9.0 (4.0–NR)	0.524
HR^−^/HER2^+^	Bone	34.0 (11.0–NR)	17.0 (4.0–36.0)	<0.001
	Liver	32.0 (10.0–49.0)	16.0 (4.0–36.0)	<0.001
	Brain	11.0 (6.0–40.0)	9.0 (3.0–17.0)	0.025
	2 of 3	26.0 (8.0–45.0)	17.0 (5.0–36.0)	0.096
	All 3	9.0 (6.0–34.0)	5.0 (2.0–12.0)	0.100
Triple‐negative	Bone	12.0 (4.0–24.0)	7.0 (3.0–15.0)	<0.001
	Liver	9.0 (5.0–18.0)	7.0 (2.0–14.0)	0.015
	Brain	8.0 (3.0–15.0)	5.0 (2.0–10.0)	0.082
	2 of 3	8.0 (4.0–14.0)	5.0 (3.0–11.0)	0.071
	All 3	5.0 (1.0–9.0)	4.0 (1.0–12.0)	0.384
Unknown	Bone	22.0 (7.0–51.0)	12.0 (3–35.0)	<0.001
	Liver	14.0 (4–34.0)	8.0 (2.0–26.0)	0.007
	Brain	8.0 (2.0–22.0)	2.0 (1–17.0)	0.073
	2 of 3	12.0 (3.0–29.0)	9.0 (2.0–28.0)	0.278
	All 3	16.0 (9.0–48.0)	2.0 (1.0–7.0)	0.013
All subtypes	Bone	33.0 (14.0–NR)	21.0 (6.0–45.0)	<0.001
	Liver	22.0 (8.0–49.0)	14.0 (4.0–33.0)	<0.001
	Brain	13.0 (4.0–34.0)	9.0 (2.0–28.0)	0.007
	2 of 3	20.0 (6.0–43.0)	15.0 (4.0–36.0)	<0.001
	All 3	10.0 (5.0–23.0)	7.0 (2.0–23.0)	0.461

HER2, human epidermal growth factor receptor 2; HR, hormone receptor; IQR, interquartile range; + Denotes positive; − denotes negative.

## Discussion

As far as we know, this work represents the first comprehensive analysis of the incidence and prognosis of patients with lung metastases at the initial diagnosis of breast cancer. In this study, we identified 4213 cases of lung metastases from patients with newly diagnosed breast cancer, accounting for 1.8% of all patients with breast cancer, 36.4% of metastatic diseases subgroup. Among them, HER2‐enriched and triple‐negative tumors had a higher percentage of lung metastases. In addition, the median survival of patients with different subtypes of lung metastases was also very heterogeneous, ranging from 11.0 months of triple‐negative subtypes to 31.0 months of HR^+^/HER2^+^ subtypes.

To date, relatively few studies have attempted to find the association between breast cancer subtypes and lung metastases. Kennecke et al. 1 reported that the cumulative rates of lung metastases in HR^+^/HER2^−^, HR^+^/HER2^+^, HR^−^/HER2^+,^ and triple‐negative subtypes were 9.1%, 17.7%, 24.1%, 15.7%, respectively, after a long‐term follow‐up of 3726 patients with early breast cancer (diagnosed from 1986 to 1992). Soni et al. 13 observed that the frequency of lung metastases in each breast cancer subtype was 17%, 14%, 25%, and 31%, respectively, in a cohort of 531 consecutive patients with advanced breast cancer. Sihto et al. 14 reported that 234 cases of distant metastases occurred in 2032 cases of breast cancer patients after follow‐up of 2.7 years. Their results indicated that the incidence of lung metastasis as first distant metastases was 8.5%, 16.3%, 22.9%, 20.8%, respectively, in luminal A, luminal B, HER2^+^‐enriched, and basal‐like subtypes 14. One of the advantages of these studies was the provision of information on the cumulative incidence of lung metastases during the natural course of the disease. In contrast to these studies, our study focused on patients presenting with lung metastases at the initial diagnosis of breast cancer. Therefore, the effect of tumor subtypes on lung metastases would not be affected by previous local and systemic treatments in our cohort.

In the present study, the median survival of patients with lung metastases was 21 months,while those who with metastases confined to lungs had a median survival of 25 months. In a retrospective analysis of M. D. Anderson Cancer Center, the median OS was 22.5 months for breast cancer patients with metastases confined to lungs treated with systemic chemotherapy 15. However, patients with metastases confined to lungs undergoing pulmonary metastasectomy had a median OS of 35–75.6 months and a 5‐year overall survival rate of 38% to 54% 5, 16, 17, 18, 19. We did not have information on lung surgery and systemic therapy for lung metastases in this cohort, so we were unable to analyze the differences in survival due to treatment. According to the tumor subtype, our study also showed important differences in OS. Patients with HR^+^/HER2^+^ subtype had the longest OS, and their risk of death was significantly lower compared to HR^+^/HER2^−^ patients. In contrast, the patients with triple‐negative subtype had the worst prognosis. Our findings were similar to previous reports on the effect of tumor subtypes on OS of patients with breast cancer 13, 20, 21, 22. Several studies showed that triple‐negative breast cancer was associated with poor prognosis 23, 24. Our findings confirmed and extended the previous reports on the effects of tumor subtypes on the prognosis of patients with breast cancer. The prognosis of patients with lung metastases observed in all tumor subtypes is quite different, confirming that breast cancer is a heterogeneous disease, even in patients with specific lung metastases.

In addition to the association between the prevalence of lung metastases and the tumor subtypes, several important correlations between lung metastases and demographics of breast cancer patients were noteworthy. Although young women were more likely to develop more aggressive breast cancer subtypes and more advanced diseases 25, 26, 27, it revealed a higher incidence of lung metastases in older patients. In addition, the percentage of lung metastases in male patients was significantly higher than that of females, although the absolute number of male breast cancer patients had not yet reached 1% of women throughout the cohort. Furthermore, black race (vs. white, OR, 1.38; 95% CI, 1.25–1.51; *P* < 0.001) had significantly greater likelihood of lung metastases at the time of diagnosis, but this association was not found in distant metastatic disease subset.

Perhaps the most interesting was that this study showed that the incidence and prognosis of lung metastases were associated with marital status and insurance status at the initial diagnosis of breast cancer, regardless of known clinical prognostic variables such as tumor subtypes, age at diagnosis, tumor grade. This confirmed and enriched previous studies which reported that the risk of cancer metastases and cancer‐related death in unmarried or uninsured patients was significantly higher than in married or insured patients 28, 29. This result emphasized the potentially significant impact of social support on breast cancer detection and survival.

Our research has some limitations. Firstly, all data were collected by the SEER program, which relied on routine collection of cancer registry data, and the incidence of lung metastases might be underestimated. Secondly, as the SEER database did not capture subsequent lung metastases during disease progression, our study was unable to incorporate subsequent lung metastases. Thirdly, we were unable to analyze the effect of lung‐directed treatment (such as lung surgery and endocrine therapy) on the prognosis of patients because these data were not available in the public SEER data set. Fourthly, while we have adjusted the effects of confounding factors such as age, insurance, and marital status, we were unable to adjust sociodemographic status to the level of the patient. Fifthly, there is no information on the number of lung lesions or the bulk of disease (i.e., lung full of tumor vs. a tiny lung met), which are important prognostic factors for patients with lung metastases. Sixthly, there may be other organs/tissue involved that are not captured by the SEER registry (i.e., adrenal glands). Finally, the distribution of the cause of death cannot be specific to lung metastases or other metastases.

Our research also had several advantages. The study was based on population‐based tumor registration in recent years, providing a generalization for the results. The sample size of this study was large enough to provide sufficient strength to explore the incidence and prognosis of lung metastases. Due to the extensive information collected by the SEER program, we were able not only to explore OS, but also to explore cancer‐specific survival. Finally, our study differed from other reports of lung metastases due to recurrence or progression of early breast cancer and was not affected by previous local and systemic treatment (which might have a potential impact on the occurrence and treatment of lung metastases), thus providing an important clinical information on the prognosis and the risk stratification of simultaneous lung metastases.

Our study provides important information on the incidence and prognosis of lung metastases in breast cancer, which is critical for designing studies to test interventions that may improve survival. In addition, the frequency of lung metastases identified in this study can be used to estimate the burden of disease, and the risk factors identified here can be used for risk‐based screening to maximize early detection of lung metastases and achieve optimal cost‐effectiveness.

## Conflict of Interest

The authors have no conflict of interest to declare.

## References

[1] Kennecke, H. , R. Yerushalmi , R. Woods , M. C. Cheang , D. Voduc , C. H. Speers , et al. 2010 Metastatic behavior of breast cancer subtypes. J. Clin. Oncol. 28:3271–3277. 20498394 10.1200/JCO.2009.25.9820

[2] Smid, M. , Y. Wang , Y. Zhang , A. M. Sieuwerts , J. Yu , J. G. Klijn , et al. 2008 Subtypes of breast cancer show preferential site of relapse. Cancer Res. 68:3108–3114. 18451135 10.1158/0008-5472.CAN-07-5644

[3] Lee, Y. T. 1983 Breast carcinoma: pattern of metastasis at autopsy. J. Surg. Oncol. 23:175–180. 6345937 10.1002/jso.2930230311

[4] Diaz‐Canton, E. A. , V. Valero , Z. Rahman , E. Rodriguez‐Monge , D. Frye , T. Smith , et al. 1998 Clinical course of breast cancer patients with metastases confined to the lungs treated with chemotherapy. The University of Texas M.D. Anderson Cancer Center experience and review of the literature. Ann. Oncol. 9:413–418. 9636832 10.1023/a:1008205522875

[5] Yhim, H. Y. , S. W. Han , D. Y. Oh , W. Han , S. A. Im , T. Y. Kim , et al. 2010 Prognostic factors for recurrent breast cancer patients with an isolated, limited number of lung metastases and implications for pulmonary metastasectomy. Cancer‐Am. Cancer Soc. 116:2890–2901. 10.1002/cncr.25054 20564396

[6] Vern‐Gross, T. Z. , J. A. Lawrence , L. D. Case , K. P. McMullen , J. D. Bourland , L. J. Metheny‐Barlow , et al. 2012 Breast cancer subtype affects patterns of failure of brain metastases after treatment with stereotactic radiosurgery. J. Neurooncol. 110:381–388. 23001361 10.1007/s11060-012-0976-3 PMC3852435

[7] Dent, R. , W. M. Hanna , M. Trudeau , E. Rawlinson , P. Sun , and S. A. Narod . 2009 Pattern of metastatic spread in triple‐negative breast cancer. Breast Cancer Res. Treat. 115:423–428. 18543098 10.1007/s10549-008-0086-2

[8] Tseng, L. M. , N. C. Hsu , S. C. Chen , Y. S. Lu , C. H. Lin , D. Y. Chang , et al. 2013 Distant metastasis in triple‐negative breast cancer. Neoplasma 60:290–294. 23373998 10.4149/neo_2013_038

[9] Rodriguez‐Pinilla, S. M. , D. Sarrio , E. Honrado , D. Hardisson , F. Calero , J. Benitez , et al. 2006 Prognostic significance of basal‐like phenotype and fascin expression in node‐negative invasive breast carcinomas. Clin. Cancer Res. 12:1533–1539. 16533778 10.1158/1078-0432.CCR-05-2281

[10] Liedtke, C. , C. Mazouni , K. R. Hess , F. Andre , A. Tordai , J. A. Mejia , et al. 2008 Response to neoadjuvant therapy and long‐term survival in patients with triple‐negative breast cancer. J. Clin. Oncol. 26:1275–1281. 18250347 10.1200/JCO.2007.14.4147

[11] Molnar, I. A. , B. A. Molnar , L. Vizkeleti , K. Fekete , J. Tamas , P. Deak , et al. 2017 Breast carcinoma subtypes show different patterns of metastatic behavior. Virchows Arch. 470:275–283. 28101678 10.1007/s00428-017-2065-7

[12] The Surveillance, Epidemiology, and End Results . (SEER) Program Research Data (1973‐2013), National Cancer Institute, DCCPS, Surveillance Research Program, Surveillance Systems Branch, released April 2016, based on the November 2015 submission. Available at http://www.seer.cancer.gov. (accessed 20 August 2017).

[13] Soni, A. , Z. Ren , O. Hameed , D. Chanda , C. J. Morgan , G. P. Siegal , et al. 2015 Breast cancer subtypes predispose the site of distant metastases. Am. J. Clin. Pathol. 143:471–478. 25779997 10.1309/AJCPYO5FSV3UPEXS

[14] Sihto, H. , J. Lundin , M. Lundin , T. Lehtimaki , A. Ristimaki , K. Holli , et al. 2011 Breast cancer biological subtypes and protein expression predict for the preferential distant metastasis sites: a nationwide cohort study. Breast Cancer Res. 13:R87. 21914172 10.1186/bcr2944 PMC3262199

[15] Rahman, Z. U. , D. K. Frye , T. L. Smith , L. Asmar , R. L. Theriault , A. U. Buzdar , et al. 1999 Results and long term follow‐up for 1581 patients with metastatic breast carcinoma treated with standard dose doxorubicin‐containing chemotherapy: a reference. Cancer‐Am. Cancer Soc. 85:104–111. 10.1002/(sici)1097-0142(19990101)85:1<104::aid-cncr15>3.0.co;2-r 9921981

[16] Yoshimoto, M. , K. Tada , S. Nishimura , M. Makita , T. Iwase , F. Kasumi , et al. 2008 Favourable long‐term results after surgical removal of lung metastases of breast cancer. Breast Cancer Res. Treat. 110:485–491. 17899365 10.1007/s10549-007-9747-9

[17] Friedel, G. , U. Pastorino , R. J. Ginsberg , P. Goldstraw , M. Johnston , H. Pass , et al. 2002 Results of lung metastasectomy from breast cancer: prognostic criteria on the basis of 467 cases of the International Registry of Lung Metastases. Eur. J. Cardiothorac. Surg. 22:335–344. 12204720 10.1016/s1010-7940(02)00331-7

[18] Chen, F. , T. Fujinaga , K. Sato , M. Sonobe , T. Shoji , H. Sakai , et al. 2009 Clinical features of surgical resection for pulmonary metastasis from breast cancer. Eur. J. Surg. Oncol. 35:393–397. 18562155 10.1016/j.ejso.2008.05.005

[19] Kycler, W. , and P. Laski . 2012 Surgical approach to pulmonary metastases from breast cancer. Breast J. 18:52–57. 22098366 10.1111/j.1524-4741.2011.01176.x

[20] Martin, A. M. , D. N. Cagney , P. J. Catalano , L. E. Warren , J. R. Bellon , R. S. Punglia , et al. 2017 Brain metastases in newly diagnosed breast cancer: a population‐based study. JAMA Oncol. 3:1069–1077. 28301662 10.1001/jamaoncol.2017.0001 PMC5824221

[21] Fallahpour, S. , T. Navaneelan , P. De , and A. Borgo . 2017 Breast cancer survival by molecular subtype: a population‐based analysis of cancer registry data. CMAJ Open 5:E734–E739. 10.9778/cmajo.20170030 PMC5621954 28951445

[22] Engstrom, M. J. , S. Opdahl , A. I. Hagen , P. R. Romundstad , L. A. Akslen , O. A. Haugen , et al. 2013 Molecular subtypes, histopathological grade and survival in a historic cohort of breast cancer patients. Breast Cancer Res. Treat. 140:463–473. 23901018 10.1007/s10549-013-2647-2 PMC3742963

[23] Foulkes, W. D. , I. E. Smith , and J. S. Reis‐Filho . 2010 Triple‐negative breast cancer. N. Engl. J. Med. 363:1938–1948. 21067385 10.1056/NEJMra1001389

[24] Reis‐Filho, J. S. , and A. N. Tutt . 2008 Triple negative tumours: a critical review. Histopathology 52:108–118. 18171422 10.1111/j.1365-2559.2007.02889.x

[25] Ahn, S. H. , B. H. Son , S. W. Kim , S. I. Kim , J. Jeong , S. S. Ko , et al. 2007 Poor outcome of hormone receptor‐positive breast cancer at very young age is due to tamoxifen resistance: nationwide survival data in Korea–a report from the Korean Breast Cancer Society. J. Clin. Oncol. 25:2360–2368. 17515570 10.1200/JCO.2006.10.3754

[26] Partridge, A. H. , M. E. Hughes , E. T. Warner , R. A. Ottesen , Y. N. Wong , S. B. Edge , et al. 2016 Subtype‐dependent relationship between young age at diagnosis and breast cancer survival. J. Clin. Oncol. 34:3308–3314. 27480155 10.1200/JCO.2015.65.8013

[27] Rosenberg, S. M. , K. J. Ruddy , R. M. Tamimi , S. Gelber , L. Schapira , S. Come , et al. 2016 BRCA1 and BRCA2 mutation testing in young women with breast cancer. JAMA Oncol. 2:730–736. 26867710 10.1001/jamaoncol.2015.5941 PMC5002892

[28] Aizer, A. A. , M. H. Chen , E. P. McCarthy , M. L. Mendu , S. Koo , T. J. Wilhite , et al. 2013 Marital status and survival in patients with cancer. J. Clin. Oncol. 31:3869–3876. 24062405 10.1200/JCO.2013.49.6489 PMC4878087

[29] Walker, G. V. , S. R. Grant , B. A. Guadagnolo , K. E. Hoffman , B. D. Smith , M. Koshy , et al. 2014 Disparities in stage at diagnosis, treatment, and survival in nonelderly adult patients with cancer according to insurance status. J. Clin. Oncol. 32:3118–3125. 25092774 10.1200/JCO.2014.55.6258 PMC4876335

